# Immunoglobulin G antibodies against *Porphyromonas gingivalis* or *Aggregatibacter actinomycetemcomitans* in cardiovascular disease and periodontitis

**DOI:** 10.1080/20002297.2017.1374154

**Published:** 2017-09-10

**Authors:** Christian Damgaard, Jesper Reinholdt, Christian Enevold, Nils-Erik Fiehn, Claus Henrik Nielsen, Palle Holmstrup

**Affiliations:** ^a^ Section of Periodontology, Department of Odontology, Faculty of Health and Medical Sciences, University of Copenhagen, Copenhagen, Denmark; ^b^ Institute for Inflammation Research, Center for Rheumatology and Spine Diseases, Rigshospitalet, Copenhagen University Hospital, Copenhagen, Denmark; ^c^ Department of Biomedicine, Faculty of Health Sciences, Aarhus University, Aarhus, Denmark; ^d^ Department of Immunology and Microbiology, Faculty of Health and Medical Sciences, University of Copenhagen, Copenhagen, Denmark

**Keywords:** Cardiovascular disease, atherosclerosis, myocardial infarction, hypertension, periodontitis, periodontal disease, antibody, immunoglobulin, risk factor, cohort study

## Abstract

**Objectives:** The aim was to elucidate whether levels of circulating antibodies to *Actinobacillus actinomycetemcomitans* and *Porphyromonas gingivalis* correlate to loss of attachment, as a marker for periodontitis and cardiovascular disease (CVD).

**Design:** Sera were collected from 576 participants of the Danish Health Examination Survey (DANHES). Immunoglobulin G antibodies against lipopolysaccharide (LPS) and protein antigens from the a, b and c serotypes of *A. actinomycetemcomitans* and *P. gingivalis* were quantified by titration in ELISA plates coated with a mixture of antigens prepared by disintegration of bacteria.

**Results:** Levels of antibodies against *P. gingivalis* (OR = 1.48) and *A. actinomycetemcomitans* (1.31) associated with periodontitis, as determined by univariable logistic regression analysis. These antibody levels also associated with CVD (1.17 and 1.37), respectively, However, after adjusting for other risk factors, including age, smoking, gender, alcohol consumption, overweight, and level of education using multivariable logistic regression analysis, only increasing body mass index (BMI; 1.09), previous smoking (1.99), and increasing age (decades) (2.27) remained associated with CVD. Increased levels of antibodies against *P. gingivalis* (1.34) remained associated with periodontitis after adjusting for other risk factors.

**Conclusions:** CVD and periodontitis were associated with levels of IgG antibodies to *P. gingivalis* or *A. actinomycetemcomitans* in univariable analyses, but only the association of *P. gingivalis* antibody levels with periodontitis reached statistical significance after adjustment for common confounders. Age, in particular, influenced this relationship.

## Introduction

Periodontitis is a highly prevalent, multifactorial inflammatory disease induced by biofilms colonizing the tooth surfaces along the gingival crevice [1]. It manifests as breakdown of the tooth-supporting tissues and, if left untreated, periodontitis may cause tooth loss [1]. The disease affects 50% of the adult population [2] and is diagnosed on the basis of clinically evident inflammation, loss of attachment associated with periodontal pocket formation, and alveolar bone loss [3]. In a recent study, the risk of myocardial infarction was significantly increased in patients with periodontitis [4]. Accordingly, recent meta-analyses demonstrated significantly increased risk of developing cardiovascular disease (CVD) in patients with periodontitis [5,6].

*Porphyromonas gingivalis* and *Aggregatibacter actinomycetemcomitans* are Gram-negative, obligate anaerobic, and facultative capnophile anaerobic rods, respectively. Both bacteria are considered residents of the periodontal biofilms associated with periodontitis [7–9]. On the basis of structurally and antigenically distinct O-antigens, six serotypes of *A. actinomycetemcomitans* (a–f) have been described, with serotype b, particularly the highly leukotoxic JP2 clone, being the dominant serotype isolated from patients with early onset aggressive periodontitis [7,10]. Interestingly, both *A. actinomycetemcomitans* and *P. gingivalis* are capable of inducing strong antibody responses exceeding those found in many other bacterial infections [11]. Elevated levels of circulating antibodies against *A. actinomycetemcomitans* and *P. gingivalis* in patients with periodontitis have often been reported [12–16]; and in a large population of adults, who participated in the third US National Health and Nutrition Examination Survey, high titers of antibodies against *P. gingivalis* were consistently associated with periodontitis [17].

In relation to CVD, increased levels of circulating antibodies towards periodontal bacteria, including both *P. gingivalis* and *A. actinomycetemcomitans*, have been associated with increased carotid intima-media thickness [18], and shown to be predictive of future myocardial infarction [19] and stroke [20]. Also, cross-sectional and case-control studies have reported positive associations between levels of antibodies against *A. actinomycetemcomitans* and *P. gingivalis* and the presence of coronary heart disease within large population-based cohorts [21–23].

According to Pussinen et al. [9], the main determinant of the systemic antibody response to *A. actinomycetemcomitans* and *P. gingivalis* is the oral carriage of these bacteria, whereas the presence or extent of periodontitis has only a modest modifying effect on the levels of antibodies against the two bacteria. Blood levels of antibodies to periodontal bacteria associated with periodontitis are stable over time [24,25], and do not change significantly following treatment [26]. However, the composition of periodontal biofilm may display variations according to the state of the disease [24–26].

We hypothesize that blood levels of antibodies to *A. actinomycetemcomitans* and *P. gingivalis* are elevated in subjects with loss of attachment (mean of 2.55 mm or more) as evidence of periodontitis, and that they correlate with the presence of CVD. Consequently, the aim was to determine, whether levels of circulating antibodies to *A. actinomycetemcomitans* and *P. gingivalis* correlate to loss of attachment, as a marker for periodontitis, and CVD. This hypothesis was tested in 576 participants in the oral health study of the Danish Health Examination Survey (DANHES) [27].

## Materials and methods

### Participants

A subgroup of 4,402 individuals (61% men and 39% women), aged 18–96 (mean 54) years, among the 18,065 volunteering participants in the DANHES study had an oral examination carried out in mobile units by three trained and calibrated dental hygienists, and filled in a lifestyle and oral health questionnaire [27]. Only one-fifth of the serum samples from the 4,402 participants from the oral part of DANHES were available. From this subpopulation, 576 subjects had both sufficient serum volume and completed questionnaires. The questionnaires consisted of open-ended questions, which the participants answered in writing on paper. As a result, incomplete questionnaires were not identified upon their submission; however, incomplete questionnaires led to exclusion of the subjects in the present study. In total, 576 participants representing 13 municipalities in Denmark including the larger Copenhagen area were included in the present study.

Furthermore, a periodontal examination including half-mouth registration (one upper and one lower quadrant randomly selected), except third molars, was completed [27]. The assessments of pocket depths (PD), bleeding on probing (BOP), and gingival margin levels were registered at six sites around each tooth. Gingival recession was registered as a negative value. Clinical attachment loss (CAL) was calculated as the sum of the PD and gingival level measurement. The manual periodontal probe (Model 8-520B; LM-Instruments, Pargas, Finland) contained pagination at 2, 4, 6, 8, 10, and 12 mm. Periodontitis was defined as a mean loss of clinical attachment of 2.55 mm or more, representing a 75th percentile of the entire study population of the oral part of DANHES [27].

CVD was self-reported in the questionnaire and defined by a history of myocardial infarction, ischemic stroke, atherosclerosis, hypertension, high blood cholesterol and/or angina pectoris, or being medically treated for CVD, as listed above. Information on risk factors was obtained from the questionnaires and used in combination with the clinical data from the oral examination.

### Serum samples

Peripheral venous blood samples were collected in Vacutainer dry tubes (BD Bioscience, Brøndby, Denmark), and left in the dark for 1 h before centrifugation at 400 *g* for 10 min. Serum was then aliquoted and stored at −80°C until further analysis.

### Ethics

The study was conducted in accordance with the Helsinki declaration and approved by the regional ethical committee, the Capital Region of Denmark (#H-C-2007-0118) and reported to the Danish Data Authority (#2007-41-15-67). Written informed consent was obtained from all participants.

### Bacterial strains

Three strains of *P. gingivalis* representing serotypes a (PGF9; originally ATCC 33277), b (PGF7; originally W83), and c (PGF75; originally HG1025), were obtained from E. Frandsen, Department of Odontology, University of Aarhus, Denmark.

Three strains of *A. actinomycetemcomitans* representing serotypes a (HK892), b (HK2108 (=Y4)), and c (HK915) were obtained from M. Kilian, Department of Biomedicine, University of Aarhus, Denmark). All three strains of *A. actinomycetemcomitans* were of the smooth colony phenotype, which allowed planktonic growth of the bacteria in liquid culture.

### Preparation of broken cell antigens

Strains of *P. gingivalis* and *A. actinomycetemcomitans* were cultured in 500 mL volumes of plaque [28] and 2×YT [29] liquid media incubated at 37°C anaerobically and in 5% CO_2_, respectively, until late exponential phase, as previously described [30,31]. The cultures were centrifuged at 6,000 *g* for 30 min at 5°C, and the isolated bacterial pellet (~2 mL) was suspended in Tris-buffered saline, pH 7.6, containing 15 mM sodium azide. The suspension was distributed in 1 mL aliquots in 2 mL Lysing Matrix A capsules (MP Biomedicals, Santa Ana, CA) and subjected to 6 × 1 min of disintegration with intermittent cooling in a FastPrep 24 homogenizer (MP Biomedicals) operated at maximal effect. After centrifugation at 10,000 *g* for 5 min, supernatants were transferred to Eppendorf tubes and centrifuged at 20,000 *g* for 5 min. The final supernatants were transferred to new tubes.

**Figure 1.**
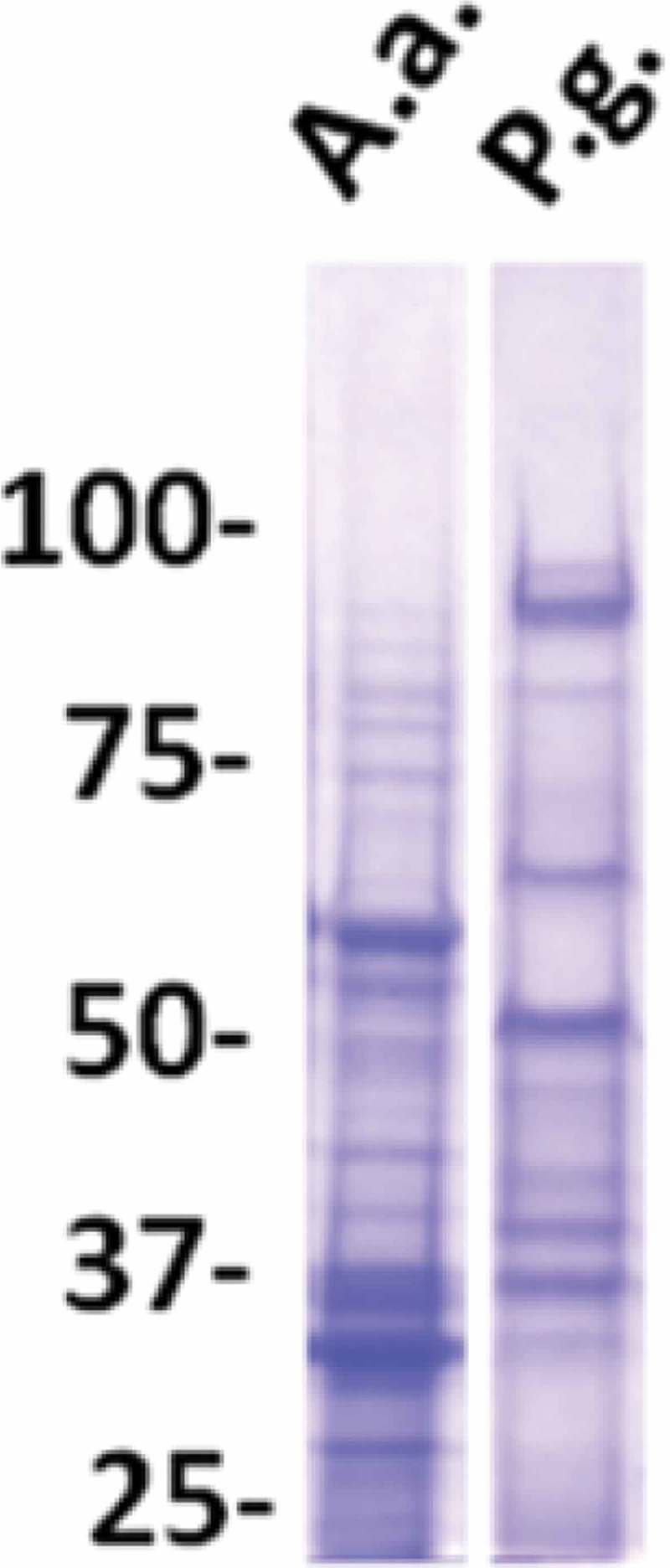
Analysis of antigen preparations from *A. actinomycetemcomitans (A.a.)* and *P. gingivalis (P.g.)* as used for coating of ELISA plates. Lanes of sodium dodecyl sulfate polyacrylamide (7%) gels were loaded with amounts of antigens equal to those used for coating of one 96-well plate. Staining with Coomassie Blue (polysaccharides and LPS) are not visible. M_r_ markers in kilo Daltons.

Antigen preparations from each of the three strains (serotypes) of *P. gingivalis* or *A. actinomycetemcomitans* were compared with respect to level of plate-coating antigens. Serial threefold dilutions of each of the three antigen preparations were coated in parallel in the same enzyme-linked immunosorbent assay (ELISA) plate (details of ELISA protocols described below). Subsequently, all wells were developed equally by incubation with a fixed dilution of a pool of three sera from strongly responding aggressive periodontitis patients [32], followed by addition of enzyme-conjugated rabbit antibody to human IgG and enzyme substrate. Based on this assay, coating antigens for use in ELISA were produced as an equal volume mixture of three antigen preparations, each at the highest dilatation still providing for saturation of the plate as judged by staining reaction in this assay. The final preparations of coating antigens contained proteins of a broad M_r_ range (Figure 1). Since natural antibodies are predominantly of the IgM isotype, potential cross-reactions with naturally occurring antibodies (against e.g. heat shock proteins) were limited by the assay’s specificity for IgG antibodies.

### Preparations of bacterial antigens in the form of whole bacteria

For use in pilot experiments, the bacterial strains cultured and isolated as described above were washed in PBS, pH 7.4, and incubated overnight in PBS containing 0.5% or 1.5% formaldehyde. After washing three times in PBS, the bacteria were suspended in PBS at a density of 10^8^ mL^−1^. For coating of ELISA plates, suspensions of individual serotypes were mixed at equal proportions.

### ELISA for quantification of bacteria-reactive antibodies

Antibodies of the IgG class to antigens of *P. gingivalis* and *A. actinomycetemcomitans* were quantified by ELISA relative to (% of) the level of antibodies in a reference serum titrated in parallel with test sera in each plate. The comparability of antibody determinations across different plates was controlled for by including in each plate the titration of a control serum. The same set of reference and control sera were used in all plates testing for antibodies against a particular bacterial species. Reference as well as control sera were pools of three sera originating from Moroccan patients with aggressive periodontitis [32]. These sera were selected on the basis of strong reactions with antigen preparations from one or more of the three contributing strains. Reference and control sera did not contain the same serum components. Before being used in the assay, reference and control sera were prediluted 1,000-fold so that the OD interval of their titration curves would match the OD interval of test sera containing less antibody activity.

ELISA protocols were carried out at room temperature throughout. Wells of polystyrene microplates (269620; Nunc, Roskilde, Denmark) were coated with 100 μL of antigen solution in 10 mM Tris-buffered saline, pH 7.6, overnight and then blocked. Tris-buffered saline containing 250 mM NaCl and 0.15% Tween 20 served as combined washing and blocking solution and as diluent. Pilot experiments had shown that uncoated plates, when blocked in this way, did not bind IgG from diluted serum or from detecting antibody preparations. Subsequently, test serum samples were applied at four serial fourfold dilutions (1:80–1:5,120), whereas reference and control sera were applied at six serial fourfold dilutions selected from results of pilot experiments. After incubation overnight and washing, wells were incubated with 100 μL of alkaline phosphatase conjugated rabbit anti-human IgG (D0336; DAKO, Glostrup, Denmark) diluted 1:3,000 for 2 h and then washed. Plates were developed with *p*-Nitrophenyl phosphate (P5994; Sigma), 1 mg mL^−1^ in 0.1 M Na-glycine buffer, pH 10.4, containing 1 mM MgCl_2_ and 1 mM ZnCl_2_. After ~45 min, optical densities (OD) at 405 nm were read with a Multiscan RC ELISA reader (Labsystems, Helsinki, Finland). A titration curve for the reference serum was fitted from a four-parameter logistic model using the Genesis LITE software package (Labsystems). Antibody concentrations in test sera were calculated as the mean of determinations based on OD readings falling within the middle 80% of the OD range of the reference serum titration curve. Control serum was prepared, so that determination of its antibody concentration could invariably be based on three OD readings representing consecutive steps of dilution. Generally, the data from a plate were considered valid if the concentration readout for the control serum did not deviate more than 20% from a value based on multiple determinations in pilot experiments.

For 180 of the serum samples, all steps of the ELISA were done manually using single and eight-channel tools for dilution and application. With these samples, dilutions of test sera, references, and controls were incubated in duplicate wells, analysis being based on mean values. The remaining 396 samples were analyzed using a laboratory robot (Biomek 3000; Beckman Coulter, CA) programmed to carry out all steps except washing, which was done with hand-held equipment (Nunc Immunowasher; Nunc, Roskilde, Denmark). To examine the functional precision of the robot, five sera representing a broad range of antibody levels against the bacterial species were titrated sixfold in one plate against both of the bacteria by the robot. Antibody determinations were highly reproducible (CV for sextuplicate antibody analysis of the five sera ranged from 3.3% to 10.7%; mean, 5.5%). Accordingly, in titrations of sera by the robot, individual dilutions were incubated in single wells.

### Evaluation of assays for bacteria-reactive antibodies

**Figure 2.**
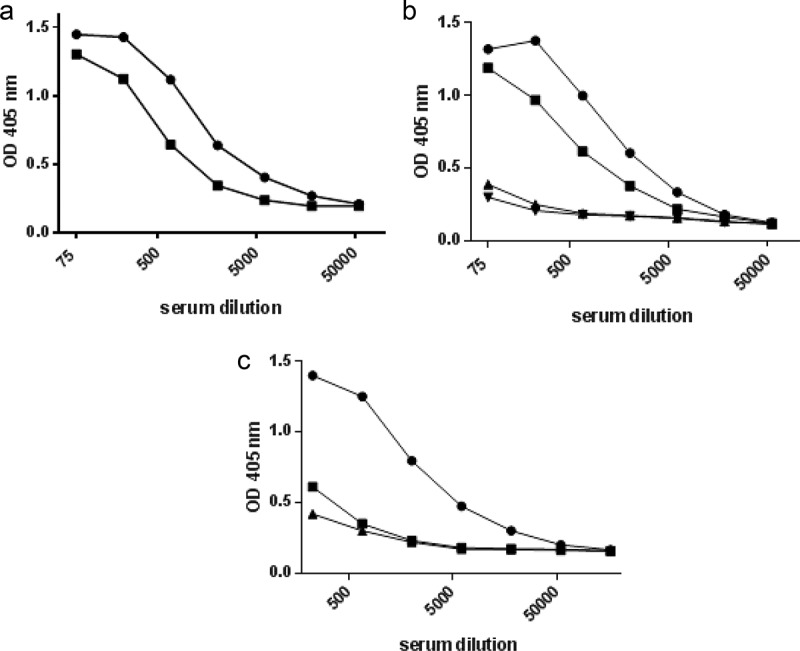
Validation of assay sensitivity and specificity to antigen preparations from *A. actinomycetemcomitans.* **A)** Homologous titration curves for serum from a patient with aggressive periodontitis colonized with the JP2 clone (serotype b) of *A. actinomycetemcomitans* in wells saturated with disintegrated serotype b bacterial antigen (●) and whole, formalin-treated serotype b bacteria (■), respectively. **B)** Comparative titration of sera from four microbiologically and clinically characterized individuals in an ELISA plate coated with an equivalent mixture of antigens from three strains of *A. actinomycetemcomitans* representing serotypes a, b, and c. The four sera were from a patient with aggressive periodontitis colonized with the JP2 clone of *A. actinomycetemcomitans* (●), a healthy individual colonized by a non-JP2 clone of *A. a*. (∎), two healthy individuals without detectable *A.a*. (▲,▼). **C)** Comparative titrations of a serum from a patient with aggressive periodontitis colonized with the JP2 clone (serotype b) of *A. actinomycetemcomitans* in ELISA wells coated with antigens from either *A. actinomycetemcomitans* serotype b (●), serotype a (∎), or serotype c (▲).

To quantify bacteria-reactive antibodies, we initially adopted a previously described assay based on coating of polystyrene ELISA plates with whole bacteria incubated overnight in PBS containing 0.5% formalin and then washed in PBS [13], modified by using formalin at 1.5% instead of 0.5% for fixation to avoid detachment of bacteria. Plates coated with broken cell antigens from *A. actinomycetemcomitans* gave stronger signals than plates coated with the whole bacterium (Figure 2(A)).

Using mixed broken cell antigens for coating, the specificity and sensitivity of the ELISA for quantification of antibodies towards *A. actinomycetemcomitans* was evaluated with sera from clinically and microbiologically characterized individuals [32]. First, serum from a juvenile aggressive periodontitis patient colonized with the JP2 clone of *A. actinomycetemcomitans* displayed a strong antibody reaction to *A. actinomycetemcomitans* antigens, whereas a lower reaction was seen in serum from a healthy individual colonized with a non-JP2 clone of this bacterium (Figure 2(B)). By contrast, sera from two healthy individuals without detectable *A. actinomycetemcomitans* colonization displayed very little antibody (Figure 2(B)).

Next, we examined the ability of our assay to detect antibodies to bacterial LPS (lipopolysaccharide), which is an immunodominant and serotype-specific antigen of *A. actinomycetemcomitans* [33]. LPS from some bacteria have been found to coat polystyrene plates poorly [34–36]. However, using broken cell antigens representing individual serotypes in ELISA, we found that serum from an aggressive periodontitis patient colonized with the JP2 clone (serotype b) of *A. actinomycetemcomitans* contained antibodies to a serotype b strain at a level more than 10 times that of antibodies to antigens from serotype a or serotype c (Figure 2(C)). This indicates that antibodies to *A. actinomycetemcomitans* detected by ELISA using broken cell antigens include antibodies to LPS, and that high levels of the antibodies reflect colonization or infection with this bacterium. Similar evaluation of the data obtained with the ELISA for antibodies to *P. gingivalis* was not possible, due to lack of sera from subjects characterized with respect to colonization with this bacterium.

### Statistics

Risk factors, which should be adjusted for in the multivariable analyses, were chosen based on available empirical data in the literature. Subsequently, univariable analyses served to confirm these risk factors. Both univariable and multivariable logistic regression analyses were performed using RStudio version 0.98.1091, R version 3.1.2. for PC (RStudio®, Boston, MA). Investigations of interactions between the selected variables were not included in the analyses due to lack of power. Possible false specification of the final regression model was assessed using the Hosmer–Lemeshow goodness of fit test. *P*-values <0.05 were considered statistically significant.

## Results

### Patient characteristics

A total of 576 participants were included in the study (Table 1). Of these, 517 participants were born in Denmark to Danish parents; 53 were born in Denmark, to parents who were born in another country; and six were born in another country than Denmark, to non-Danish parents. The study population consisted of 46% women and 54% men, with a mean age of 54 years. For further details, see Table 1.

**Table 1.** Distribution of study population exposure- and covariables by cardiovascular disease (CVD) and periodontitis.

	All	CVD	Periodontitis
All	576 (100%)	200 (35%)	208 (36%)
Age (mean [SD]), years	54 (14)	62 (10)	61 (9)
Sex			
Male	311 (54%)	95 (31%)	104 (33%)
Female	265 (46%)	105 (40%)	104 (39%)
Smoking status			
Never	252 (44%)	68 (27%)	60 (24%)
Previous	221 (38%)	106 (48%)	96 (43%)
Current	103 (18%)	26 (25%)	52 (50)
Alcohol consumption, units/week			
None	84 (15%)	30 (36%)	27 (32%)
1–7 (women)/1–14 (men)	334 (58%)	110 (33%)	109 (33%)
8–14 (women)/15–21 (men)	101 (18%)	36 (36%)	39 (39%)
>14 (women)/>21 (men)	57 (10%)	24 (42%)	33 (58%)
Diabetes*			
HbA_1c_ <6.5%	555 (96%)	186 (34%)	199 (36%)
HbA_1c_ ≥6.5%	21 (4%)	14 (67%)	9 (43%)
ISCED			
≤10 years	91 (16%)	37 (41%)	44 (48%)
11–12 years	117 (20%)	41 (35%)	52 (44%)
13–14 years	126 (22%)	33 (26%)	36 (29%)
≥15 years	242 (42%)	89 (37%)	76 (31%)
BMI (mean [SD])	25 (4)	26 (4)	26 (4)
CAL mm (mean [SD])	2.52 (1.02)	2.72 (1.06)	3.48 (1.09)
Antibody titers (median [range])			
*A. actinomycetemcomitans*	5 (0–1011)	8 (0–1,011)	7 (0–1,011)
*P. gingivalis*	82 (2–25,600)	104 (5–25,600)	158 (7–25,600)

BMI, body mass index; CAL, clinical attachment level; CVD, cardiovascular disease; HbA_1c_, glycosylated hemoglobin; ISCED, International Standard Classification of Education; SD, standard deviation. *Defined as HbA_1c_ ≥6.5 in peripheral blood.

### Periodontitis

In the present study, periodontitis, defined as a mean clinical loss of attachment of 2.55 mm or more, representing the 75 percentile of the entire study population of the oral part of DANHES [27], was significantly associated with IgG antibodies against both *P. gingivalis* (OR = 1.48 [95% CI: 1.31–1.67]) and *A. actinomycetemcomitans* (1.31 [1.13–1.52]) using univariable logistic regression analysis (Table 2).

**Table 2.** Periodontitis: univariable logistic regression analyses.

	p-value	OR (95% CI)
Age (decades)	**5.6 × 10^−19^**	**2.25 (1.89–2.70)**
Sex (male)	0.150	1.29 (0.91–1.81)
Smoking status		
Previous smoker	**7.5 × 10^−6^**	**2.46 (1.66–3.66)**
Current smoker	**1.6 × 10^−6^**	**3.26 (2.02–5.31)**
Alcohol consumption, units/week		
0	0.930	0.98 (0.58–1.62)
8–14 (women)/15–21 (men)	0.270	1.30 (0.81–2.05)
>14 (women)/>21 (men)	**3.6 × 10^−4^**	**2.84 (1.61–5.08)**
Diabetes*	0.510	1.34 (0.54–3.23)
ISCED group		
1 (≤10 years)	**0.004**	**2.04 (1.25–3.35)**
2 (11–12 years)	**0.016**	**1.75 (1.11–2.75)**
3 (13–14 years)	0.580	0.87 (0.54–1.39)
BMI	0.057	1.04 (1.00–1.09)
Log *A. actinomycetemcomitans* Abs	**4.3 × 10^−4^**	**1.31 (1.13–1.52)**
Log *P. gingivalis* Abs	**2.0 × 10^−10^**	**1.48 (1.31–1.67)**
CVD	**0.001**	**1.79 (1.26–2.55)**

Bold values indicate statistical significance (p < 0.05). Abs, serum IgG antibodies; BMI, body mass index; CVD, cardiovascular disease; ISCED, International Standard Classification of Education; log, logarithm transformed. *Defined as HbA_1c_ ≥6.5 in peripheral blood.

Moreover, by univariable logistic regression analyses, periodontitis was associated with increasing age (decades) (2.25 [1.89–2.70]), with CVD and/or medical treatment for CVD (1.79 [1.26–2.55]), and weekly alcohol consumption of more than 14 units of alcohol for women and 21 for men (2.84 [1.61–5.08]). Current and previous smokers both exhibited increased risk of having developed periodontitis (current smokers: 3.26 [2.02–5.31] and previous smokers: 2.46 [1.66–3.66]). Also, less than 12 years of education were associated with periodontitis (ISCED group 1: 2.04 [1.25–3.35], and ISCED group 2: 1.75 [1.11–2.75]) (Table 2).

However, following adjustment for common risk factors, only smoking (current smoker: 4.72 [2.61–8.69] and previous smoker: 2.21 [1.39–3.55]), increasing age (decades; 2.35 [1.90–2.96]), and *P. gingivalis*-reactive IgG remained significantly associated with periodontitis (1.34 [1.17–1.54]) in the multivariable logistic regression analyses (Table 3).

**Table 3.** Periodontitis: multivariable logistic regression analyses.

	p-value	OR (95% CI)
Age (decades)	**3.96 × 10^−14^**	**2.35 (1.90–2.96)**
Sex (male)	0.442	0.84 (0.54–1.30)
Smoking status		
Previous smoker	**0.001**	**2.21 (1.39–3.55)**
Smoker	**3.86 × 10^−7^**	**4.72 (2.61–8.69)**
Alcohol consumption, units/week		
0	0.936	0.98 (0.53–1.78)
8–14 (women)/15–21 (men)	0.959	0.99 (0.57–1.69)
>14 (women)/>21 (men)	0.137	1.65 (0.86–3.22)
Diabetes*	0.476	0.69 (0.24–1.92)
ISCED group		
1 (≤10 years)	0.222	1.42 (0.81–2.52)
2 (11–12 years)	0.351	1.30 (0.75–2.26)
3 (13–14 years)	0.850	1.06 (0.60–1.85)
BMI	0.812	1.01 (0.95–1.06)
CVD/CVD medicine	0.188	0.74 (0.47–1.16)
Log *A. actinomycetemcomitans* Abs	0.812	1.02 (0.85–1.23)
Log *P. gingivalis* Abs	**1.94 × 10^−5^**	**1.34 (1.17–1.54)**

Bold values indicate statistical significance (p < 0.05). Abs, serum IgG antibodies; BMI, body mass index; CVD, cardiovascular disease; ISCED, International Standard Classification of Education; log, logarithm transformed. *Defined as HbA1c ≥6.5 in peripheral blood.

### Cardiovascular disease

In the present study, we broadly defined CVD upon self-reported previous cardiovascular events, consumption of antihypertensive and/or anticoagulant medication, or based on elevated blood pressure measures in the clinical examination performed in conjunction with the DANHES study.

Using univariable logistic regression analyses, both mean loss of attachment of 2.55 mm or more (1.79 [1.26–2.55]) and presence of IgG antibodies to *P. gingivalis* (1.17 [1.05–1.31]) or *A. actinomycetemcomitans* (1.37 [1.18–1.60]) were significantly associated with CVD. Known risk factors for CVD, such as increasing body mass index (BMI) and diabetes, were also associated with CVD in this study (Table 4). Previous smokers were more likely to have CVD (2.49 [1.70–3.67]), but, surprisingly, current smokers did not exhibit a significantly increased risk of CVD; this may be explained by the age distribution of current versus previous smokers, the latter being on average 6 years older. Moreover, former smokers were 7 years older on average than never-smokers. Alcohol consumption was not associated with increased prevalence of CVD in the present study population (Table 4). Finally, individuals with 11–12 years of education were less likely to have CVD (0.61 [0.38–0.96]).

**Table 4.** Cardiovascular disease: univariable logistic regression analyses.

	p-value	OR (95% CI)
Age (decades)	**4.4 × 10^−19^**	**2.31 (1.93–2.79)**
Sex (male)	0.023	1.49 (1.06–2.11)
Smoking status		
Previous smoker	**3.0 × 10^−6^**	**2.49 (1.70–3.67)**
Current smoker	0.740	0.91 (0.53–1.53)
Alcohol consumption, units/week		
0	0.630	1.13 (0.68–1.86)
8–14 (women)/15–21 (men)	0.610	1.13 (0.70–1.79)
>14 (women)/>21 (men)	0.180	1.48 (0.83–2.62)
Diabetes*	**0.004**	**3.97 (1.62–10.62)**
ISCED group		
1 (≤10 years)	0,520	1.18 (0.72–1.92)
2 (11–12 years)	0,750	0.93 (0.58–1.47)
3 (13–14 years)	**0.042**	**0.61 (0.38–0.97)**
BMI	**9.6 × 10^−6^**	**1.11 (1.06–1.16)**
Log *A. actinomycetemcomitans* Abs	**4.1 × 10^−5^**	**1.37 (1.18–1.60)**
Log *P. gingivalis* Abs	**0.006**	**1.17 (1.05–1.31)**
Periodontitis†	**0.001**	**1.79 (1.26–2.55)**

Bold values indicate statistical significance (p < 0.05). Abs, serum IgG antibodies; BMI, body mass index; CVD, cardiovascular disease; ISCED, International Standard Classification of Education; log, logarithm transformed. *Defined as HbA_1c_ ≥6.5 in peripheral blood. †Defined as mean loss of attachment of ≥2.55 mm.

After adjustment for common risk factors such as age, smoking, gender, alcohol consumption, overweight, and level of education in a multivariable logistic regression analysis, CVD was no longer significantly associated with levels of IgG antibodies against *P. gingivalis* (1.04 [0.91–1.18]) or *A. actinomycetemcomitans* (1.13 [0.94–1.36]), nor with periodontitis (0.76 [0.48–1.15]) (Table 5).

**Table 5.** Cardiovascular disease: multivariable logistic regression analyses.

	p-value	OR (95% CI)
Age (decades)	**5.16×10^−14^**	**2.27 (1.85–2.84)**
Gender (men)	0.683	1.09 (0.71–1.67)
Smoking status		
Previous smoker	**0.002**	**2.00 (1.28–3.15)**
Current smoker	0.941	1.02 (0.55–1.89)
Alcohol consumption, units/week		
0	0.278	1.39 (0.76–2.50)
8–14 (women)/15–21 (men)	0.610	0.87 (0.50–1.50)
>14 (women)/>21 (men)	0.809	1.08 (0.56–2.09)
Diabetes*	0.059	2.84 (0.99–8.80)
ISCED group		
1 (≤10 years)	0.119	0.63 (0.35–1.12)
2 (11–12 years)	**0.038**	**0.56 (0.32–0.96)**
3 (13–14 years)	0.068	0.60 (0.34–1.03)
BMI	**0.001**	**1.09 (1.04–1.15)**
Periodontitis†	0.218	0.76 (0.48–1.18)
Log *A. actinomycetemcomitans* Abs	0.183	1.13 (0.94–1.36)
Log *P. gingivalis* Abs	0.605	1.04 (0.91–1.18)

Bold values indicate statistical significance (p < 0.05).

Abs, serum IgG antibodies; BMI, body mass index; CVD, cardiovascular disease; ISCED, International Standard Classification of Education; log, logarithm transformed. *Defined as HbA1c ≥6.5 in peripheral blood. †Defined as mean loss of attachment of ≥2.55 mm.

Risk factors commonly associated with CVD, such as increased BMI (1.09 [1.04–1.15]), status as a previous smoker (2.00 [1.28–3.15]), or increasing age (decades; 2.27 [1.85–3.15]) remained associated with CVD in the multivariable logistic regression analysis (Table 5). Moreover, 11–12 years of education or more remained associated with less CVD after adjustment for other risk factors (0.56 [0.32–0.96]).

## Discussion

In the present study we have examined the associations between periodontitis, CVD, and concentration of IgG antibodies to *P. gingivalis* and *A. actinomycetemcomitans* in a cohort of 576 subjects (Table 1), which is a large cohort in comparison to the existing literature on this topic [9,19–23]. As determinant of periodontitis, we used a mean loss of attachment of at least 2.55 mm [27,37,38]. This may not be a precise assessment measure of the disease processes associated with periodontitis, because it only includes evidence of past tissue degeneration and not the current state of the disease.

High titers of antibodies against both *P. gingivalis* and *A. actinomycetemcomitans* were associated with periodontitis using univariable analyses. However, only *P. gingivalis*-reactive antibody titers remained significantly associated with periodontitis, when the data were adjusted for common risk factors in multivariable logistic regression analyses. This finding substantiates the positive and independent association of serum antibody levels against *P. gingivalis* with periodontitis, which then supports the premise, that serum antibody levels against *P. gingivalis* can be used for investigating potential associations between periodontitis and other diseases such as CVD. Others have also successfully used broken cell antigens for titration of antibodies in serum *P. gingivalis*-positive and -negative subjects by ELISA [39].

Periodontitis was associated with self-reported previous cardiovascular events, antihypertensive and/or anticoagulant medication, or high blood pressure in univariable analyses. However, these associations did not reach statistical significance after adjusting for common risk factors.

In a previous study, Beck et al. found lack of association between periodontitis and coronary heart disease, whereas antibody response to several oral organisms, including *P. gingivalis* and *A. actinomycetemcomitans*, did associate with periodontitis in agreement with our findings for antibodies against *P. gingivalis* [18]. The authors suggested that the extent of bacterial exposure may be more important for systemic health than the burden of periodontitis.

We found that high levels of serum antibodies against *P. gingivalis* and *A. actinomycetemcomitans* were associated with CVD. However, after adjustment for common risk factors, the association between CVD and antibodies against *P. gingivalis* and *A. actinomycetemcomitans* lost significance. Adjustment for increasing age, in particular, influenced the association observed in the univariable analyses. Previous studies have consistently adjusted for age when investigating associations between antibody levels against *P. gingivalis* and *A. actinomycetemcomitans*, periodontitis, and CVD [19–23]; our data support the hypothesis that increasing age is an independent risk factor for loss of attachment.

The incidence and magnitude of bacteremia are increased in patients with periodontitis compared with periodontally healthy subjects [40–43]. The biological background for the association between periodontitis and CVD has been related to dissemination of bacteria, such as *P. gingivalis* and *A. actinomycetemcomitans* and their products during everyday oral procedures including chewing, tooth brushing, and flossing. Another potential pathogenic pathway is spillover of inflammatory mediators from periodontal tissues to the circulation during such everyday oral procedures [40]. Notably, both viable oral bacteria and DNA from oral bacteria have been isolated in atheromatous plaques distant from the oral cavity [44–46]. Concomitant changes in endothelial permeability promote the entry and retention of cholesterol-containing low-density lipoprotein particles in the artery wall, which may aggravate atherosclerotic lesions and thus the risk of cardiovascular events [47]. Moreover, bacteria in the circulation may induce the release of pro-coagulant factors by endothelial cells, or even apoptosis, causing rupture of the atherosclerotic plaque [48].

*A. actinomycetemcomitans* is particularly implicated in the pathogenesis of periodontitis in populations of North African descent. However, *A. actinomycetemcomitans* and *P. gingivalis* can also be found in low numbers in the periodontal biofilms of periodontally healthy subjects [1]. On that basis, we expected low titers of antibodies against *A. actinomycetemcomitans* in the current study population. Nevertheless, we did find antibodies against *A. actinomycetemcomitans* with varying titers (Table 1) in our cohort, despite consisting of almost only Caucasians. However, these did not associate with periodontitis after adjustment for common risk factors. IgG antibodies against *P. gingivalis* on the other hand correlated with periodontitis defined as a mean clinical loss of attachment of 2.55 mm or more (Table 3).

In conclusion, IgG antibody levels against *A. actinomycetemcomitans* and *P. gingivalis* were associated with CVD; however, when other risk factors were taken into account, statistical significance was lost. IgG antibodies against *P. gingivalis* correlated with mean loss of attachment, which not only substantiates the role of *P. gingivalis* in the pathogenesis of periodontitis, but also suggests that serum IgG antibodies against *P. gingivalis* may serve as biomarkers for periodontitis in studies of associations between periodontitis and systemic diseases.

## Highlights

- Periodontitis is associated with levels of IgG antibodies to both *P. gingivalis* and *A. actinomycetemcomitans* in univariable analyses.
- Cardiovascular disease is associated with levels of IgG antibodies to both *P. gingivalis* and *A. actinomycetemcomitans* in univariable analyses.
- Levels of IgG antibodies to *P. gingivalis* are significantly associated with periodontitis in multivariable logistic regression analyses adjusted for common confounders such as age, smoking, gender, and BMI.
