# Corrigendum

**DOI:** 10.1080/20002297.2018.1433122

**Published:** 2018-01-31

Damgaard C, Reinholdt J, Enevold C, et al. Immunoglobulin G antibodies against *Porphyromonas gingivalis* or *Aggregatibacter actinomycetemcomitans* in cardiovascular disease and Periodontitis. J Oral Microbiol. 2017;9(1): 1374154.

https://doi.org/10.1080/20002297.2017.1374154

When the article was published online, the organism name ‘*Aggregatibacter actinomycetemcomitans’* in the abstract section has been incorrectly published as *‘Actinobacillus actinomycetemcomitans’*. This has now been corrected.

